# On the Capacity of the Peak-Limited and Band-Limited Channel

**DOI:** 10.3390/e26121049

**Published:** 2024-12-03

**Authors:** Michael Peleg, Shlomo Shamai

**Affiliations:** Department of Electrical and Computer Engineering, Technion—Israel Institute of Technology, Haifa 3200003, Israel

**Keywords:** peak power, capacity, AWGN, band-limited, entropy

## Abstract

We investigate the peak-power limited (PPL) Additive White Gaussian Noise (AWGN) channels in which the signal is band-limited, and its instantaneous power cannot exceed the power *P*. This model is relevant to many communication systems; however, its capacity is still unknown. We use a new geometry-based approach which evaluates the maximal entropy of the transmitted signal by assessing the volume of the body, in the space of Nyquist-rate samples, comprising all the points the transmitted signal can reach. This leads to lower bounds on capacity which are tight at high Signal to Noise Ratios (SNRs). We find lower bounds on capacity, expressed as power efficiency, that were higher than the known ones by a factor of 3.3 and 8.6 in the low-pass and the band-pass cases, respectively. The gap to the upper bounds is reduced to a power ratio of 1.5. The new bounds are numerically evaluated for FDMA-style signals with limited duration and also are derived in the general case as a conjecture. The penalty in power efficiency due to the peak power constraint is roughly 6 dB at high SNRs. Further research is needed to develop effective modulation and coding for this channel.

## 1. Introduction

Signals transmitted by wireless communication systems are limited in their power due to the limited capabilities of practical hardware. Two types of power limits were studied already by Shannon [1], namely the average power, which is the long-term average over all the transmitted sequences, and the peak power, which is the maxima of the instantaneous power over the whole transmission. We investigated the peak-power limited (PPL) Additive White Gaussian Noise (AWGN) channels in which the signal is band-limited and its instantaneous power cannot exceed the power *P*. This model is relevant to many systems in which the peak power is limited by the power amplifier at the transmitter. The model became even more important with the introduction of Digital Pre-Distortion (DPD), e.g., in [2,3], which linearizes the power amplifier up to its maximal transmit power, thus causing it to perform as an ideal soft limiter. Clearly, the capacity limits of this channel are of major practical interest, e.g., the optimization in Section 3.6 of [3], and the interesting peak-power reducing schemes in [4]. Indeed, Shannon already analyzed this channel and presented lower and upper bounds on capacity in [1]. With the exact capacity of the classical Average Power Limited (APL) channel found by Shannon [1] and used widely for tens of years, the PPL channel capacity was studied only sparsely, yielding lower and upper bounds on capacity with a wide gap in between (see [5,6] and Table 1). We think the reason is the difficulty in analyzing this channel as suggested already in [1]. The importance of limiting the peak power is reflected in many works analyzing and reducing the Peak to Average Power Ratio (PAPR), e.g., [7,8,9]. The impact of the peak power limit has been classical in many communications settings since the start of the wireless communication era, and it is relevant to a variety of practical communications, e.g., modulation and coding over fading channels in [4]. The problem investigated here is related to communication over the Constrained Gaussian Channel (CGC) [10,11,12], in which a wideband peak-limited signal is fed into a transmit filter in the transmitter. We show below that the capacity of the CGC is an upper bound of the capacity of the PPL channel. The review in [13] presents and categorizes a wide range of modulation schemes with different types of peak limits, including the CGC and the PPL models.

There are two known types of upper bounds on the capacity of the PPL channel. The first one uses the result of [14] on the power spectral density (PSD) of unit processes, which are the inputs to the CGC, to derive the upper bounds of capacity [12] of the CGC which are also valid for the PPL channel. In [15], the approach is specified for the PPL channel, gaining additional insights. The second type of upper bounds releases the constraint on the peak power by applying it only to samples of the signal taken at the Nyquist sampling rate and then computing capacity based on the Nyquist-rate samples being sufficient statistics of the received signals. We denote this approach here as the “sampled discrete analysis”. This is introduced in [1] and used in [5] utilizing the capacity of the scalar peak-limited channel derived in [16]. The known lower bounds on capacity are obtained by achievability schemes based on identically and independently distributed (i.i.d.) symbols with optimized pulse shapes (see [1,5,6]).

In this work, we provide numerical evaluation of a lower bound on capacity which is valid for cyclic prefix-assisted Frequency Domain Equalization (CP-FDE) signalling of lengths of up to 100 channel symbols. The CP-FDE signals are not strictly band-limited because they are limited in time; however, they are practically band-limited in the sense of having zero inter-channel interference between users if the rules for cyclic prefixes are adhered to, thus enabling the assignment of adjacent users to channels with no frequency gaps in between. This is applied, for example, in the multiuser uplink of the Long-Term Evolution (LTE) mobile communications system using the Single-Carrier FDMA (SC-FDMA) [17]. Furthermore, we present a lower bound on the capacity of the general PPL channel, which is conjecture due to the two analytical approximations used. We provide lower bounds on capacities improved about 5 dB and more relative to [5,6]. We show that our lower bounds are tight at asymptotically high Signal to Noise Ratios (SNRs), while it is well known [1] that, at very low SNRs, the PPL capacity approaches that of the APL. We investigate first the real-valued channels which model low-pass signals and then the complex-valued channels which model the bandpass signals relevant to radio frequency communications.

The lower bounds in [1,5,6] were obtained using independently and identically distributed (i.i.d.) symbols. Our new approach utilizes dependencies between symbols to increase capacity while meeting the peak power constraint. Modern efforts at PAPR reduction use diverse methods, frequently adapting the transmission per each individual information sequence resembling coding, e.g., [9]. We found that the signals emerging in our new bounds utilize only a small subset of possible symbol sequences selected by the peak power constraint resembling, in a way, coding, in which only a small subset of all possible binary sequences selected by the parity check matrix are valid codewords.

Our new approach is geometry-based; it evaluates the maximal entropy of the transmitted signal by assessing the volume of the body comprising all the points the transmitted signal can reach in the space of Nyquist-rate samples. This is related to the technique introduced in [18] over the CGC. A preprint version of this paper containing additional figures is available in [19].

Notation: Log is the natural logarithm unless stated otherwise. Differential entropy is denoted by *h*; *E* denotes the statistical expectation. The *N*-dimensional vector space of real variables is denoted *R^N^*. The Probability Density Function (PDF) of *x* is *p_x_*(*x*) or *p*(*x*). Bold italic letters denote vectors. A full list of symbols, variables and abbreviations is provided in Appendix C.

## 2. System Model

**Figure 1:**
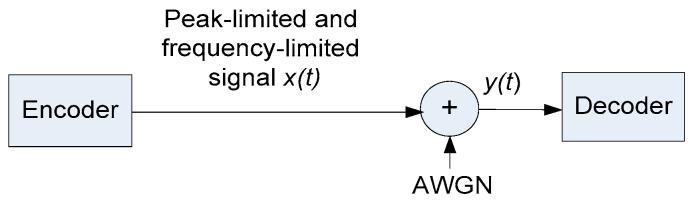
System model of the PPL channel.

We begin with the real-valued channel. The system is presented in Figure 1. The encoder produces a real-valued low-pass signal *x*(*t*) in the frequency band |*f*| < *B*. The signal is peak-limited, that is, $\left|x\left(t\right)\right|\leq \sqrt{P}\;\mathrm{for}\;\mathrm{all}\;t$. The signal passes an AWGN channel and is decoded. The channel output *y* is

(1) $$y\left(t\right)=x\left(t\right)+n\left(t\right)$$

where *n*(*t*) is a white Gaussian noise with power spectral density *N*_0_ (0.5*N*_0_, two-sided) and power ${\sigma_{n}}^{2}=N_{0}B$. The Nyquist interval is *T = 0.5/B.* The Signal to Noise ratio is defined as $\rho =\frac{P}{BN_{0}}\;.$ The Nyquist-rate sample *x*(*t*) is denoted by the vector ***x*** = (*x*_1_ *… x_n_ … _N_*) of length *N.* We seek bounds on the capacity *C*, which is the maximal Mutual Information (MUI) denoted *$I\left(x;y\right)\;$*per Nyquist interval, $C=\frac{1}{N}I\left(x;y\right)$. In the following, we shall analyze the MUI mostly over finite intervals *N*. As shown in [1], to achieve reliable communication with bitrate approaching the capacity, either coding over many such intervals is required or *N* has to be taken to its infinite limit.

The capacity in bits per Nyquist interval of a similar APL system in which the peak power limit *P* is replaced by the average power limit *P* is the following famous equation [1]:

(2) $$C_{a}=0.5\log_{2}\left(\frac{P}{N_{0}B}+1\right)$$

As stated in the Introduction, the capacity of the CGC is an upper bound of the capacity of the PPL channel. The proof is through Lemma 1 of [10], which implies that any signal permitted at the channel input by the PPL model is also valid under the CGC model.

## 3. Analysis

### 3.1. General Analysis

The lower bound on capacity can be obtained from the differential entropy *h*(***x***) of the transmitted signal ***x*** via the Entropy Power Inequality (EPI), e.g., [20], as performed, e.g., in [10]. The derivation is presented in Appendix A for completeness. The lower bound γ on the power loss ratio of the PPL channel relative to that of APL channel (2) valid at all SNRs is defined in the sense of (3):

(3) $$\;\;C\geq 0.5\log_{2}\left(\frac{\gamma \cdot P}{N_{0}B}+1\right)$$

It is shown in Appendix A that the following holds:

(4) $$\gamma =\frac{P^{e}}{P}\leq 1$$

where *P^e^* denotes the entropy power of ***x***, defined as

(5) $$P^{e}=\frac{1}{2\pi e\;}\;\cdot \;e^{\frac{2}{N}h\left(x\right)}$$

The ratio γ is the pre-SNR factor in [6] and it is unity for the APL channel, leading from (3) to (2). The value of γ in (5) is a function of the interval length *N*. The influence of *N* is moderate and treated in the sections below.

If the transmitted vector of the *N* Nyquist-rate samples ***x*** is confined to some region of *R^N^* with a volume *V_x_*, then the maximal *h*(***x***) is the logarithm of the volume *V_x_* and is achieved by the uniform distribution of ***x*** over *V_x_*. As shown in Appendix A, under uniform distribution we have

(6) $$P^{e}=\frac{1}{2\pi e}V_{x}^{\frac{2}{N}}$$

Combining this with (4) yields

(7) $$\gamma =\frac{V_{x}^{\frac{2}{N}}}{2\pi e}$$

evaluated for signals with peak power *P* = 1 (see Appendix A).

To provide an upper bound on *γ*, the peak power limit can be applied on the Nyquist-rate samples only and not on the signal in between, as performed in [5], yielding $\gamma =\frac{2}{\pi e}$; see Appendix A for further explanation.

The lower bounds (3) and (7), which are valid for all SNRs, were shown by [16] to be tight at asymptotically high SNRs for the one-dimensional system analyzed in [16]; they are tight at asymptotically high SNRs in our case too, as shown below in Equation (13).

We follow the method of evaluating the volume *V_x_* presented in [18]. As in [18], all the peak-limited signals form a convex body where convex means that for any pair ***x***_1_, ***x***_2_ in *V_x_*, any linear combination of the two vectors *a**x***_1_ + *b**x***_2_, with *a*, *b* being positive and *a*_1_ + *a*_2_ = 1, is in *V_x_*. This holds in our case since the absolute value of the linear combination is upper-bounded as

$$\left|ax_{1}\left(t\right)+bx_{2}\left(t\right)\right|\leq a\left|x_{1}\left(t\right)\right|+b\left|x_{2}\left(t\right)\right|;\;a+b=1,\;a,b>0.$$

We seek the volume *V_x_* of the *N*-dimensional convex set ***x***, which includes the origin. We denote by *r* the Distance From the Origin (DFO) to the set surface along the direction of some vector ***x***. We denote *r* as the DFO, while in [21] the term radial function is used. We denote the PDF of *r* by *p*(*r*) for angles *θ* from the origin to the surface selected randomly and uniformly over the *N* − 1-sphere. Then, the volume is

(8) $$V_{x}=E_{\theta }\left(V_{N}^{u}\cdot r^{N}\right)$$

where *E_θ_* denotes expectation with respect to *θ* distributed uniformly over the *N* − 1-sphere as defined above and $V_{N}^{u}$ denotes the volume of the *N*-dimensional ball with unit radius. The volume equation is equivalent to proposition 1.13 in [21], where *r* is denoted radial function. In [21], the expectation is replaced by an integral over all directions. This is equivalent since, in our calculation, the directions are uniformly distributed over the unit *N* − 1-sphere. The conversion between (8) here and proposition 1.13 in [21] also utilizes the relation $S_{N-1}^{u}=N\cdot V_{N}^{u}$, where $S_{N-1}^{u}\;$is the area of the unit *N* − 1 sphere. The expression in [21] is stated and valid for star-shaped bodies which are a generalization of convex bodies including the origin used in this work.

**Figure 2:**
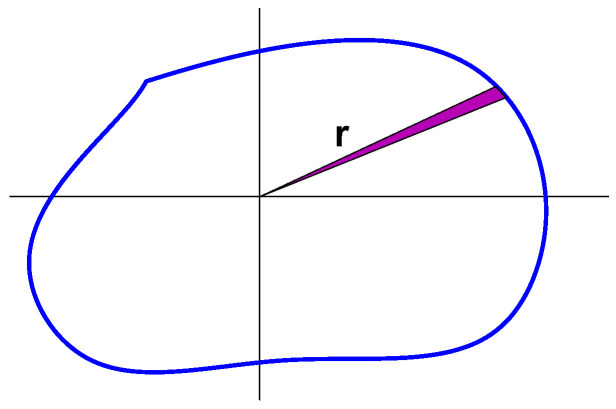
Illustration of the estimation of the volume of a convex set.

To estimate the volume, we drew random vectors ***x*** with angles *θ* spread uniformly over the *N* − 1 sphere, calculated *r* for each, substituted them into (8) and computed the average. The random vectors were generated by a method taken from [22], which uses vectors of independent random Gaussian components. The estimation of *V_x_* is illustrated in Figure 2 in two dimensions.

This approach is applied to our problem through the following:

1. Generating, numerically or analytically, a signal at a random direction distributed uniformly on the *N* − 1 sphere. This is conducted by drawing all the samples independently from a Gaussian distribution.
2. Scaling the generated signal to observe the peak limit of *x*(*t*) at any time and sampling it at Nyquist rate to obtain the vector ***x***. The resulting length *L* of ***x*** determines the local radius *r* (DFO) in this direction.
3. Averaging as defined in (8) over many directions yields the volume of the convex body.

The analysis needs to be careful because, in most cases, the peak of the signal before normalization will be very large (maximum of many Gaussian variables) and the volume will be determined mostly by the minority of signals in which the peak is moderate. This minority vanishes with growing *N*. To address this issue, we shall perform the analysis on blocks of length of *NT* and then estimate the limit as *N* approaches infinity.

### 3.2. Sampled Discrete Analysis

The analysis of maxima of continuous Gaussian processes is difficult and will require approximations [23]. For an initial analysis, we shall peak-limit the signal using only the *N* Nyquist-rate samples. This is the same as the problem of a sampled system using discrete power-limited symbols treated in [1,5,6] and serves to the evaluate upper bound on γ and to develop our analysis method. The analysis is generalized further below to continuous signals, which are the principal subject of this work. We need the PDF of

$$z=\underset{i∋\left\{1\ldots N\right\}}{\max}\left|x_{i}\right|$$

This is available from the theory of order statistic, e.g., [24,25]. From [24], Equation (2.1.6), we have

$$p_{z}\left(z\right)=N\cdot F_{\left|x\right|}{\left(z\right)}^{N-1}\cdot p_{\left|x\right|}\left(z\right)$$

where *p* and *F* denote the PDF and Cumulative Distribution Function (CDF), respectively.

For Gaussian *x* of unity power *P* with CDF,

$$F_{X}\left(x\right)=P\left(x\leq X\right)=1-Q\left(x\right)$$

where Q denotes the Q-function. This yields, after few standard steps,

(9) $$p_{Z}\left(z\right)=N{\left[1-2Q\left(z\right)\right]}^{N-1}\frac{2}{\sqrt{2\pi }}e^{-\frac{z^{2}}{2}}$$

**Figure 3:**
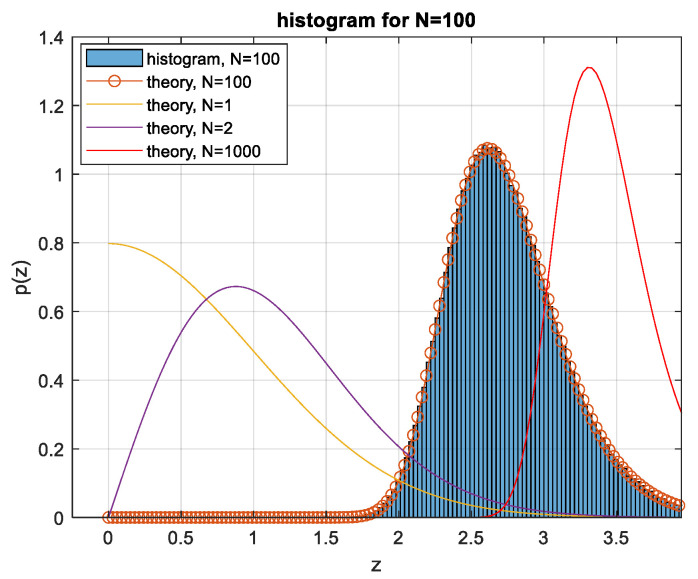
The PDF of the maxima z of absolute values of *N* Gaussian random variables *x_i_*.

The distribution *p(z)* is presented for various *N* values and verified by simulation in Figure 3.

To evaluate the DOF *r*, we need the PDF of the vector ***x*** of the Gaussian variables when the maxima *z* of the absolute value of its elements is given. This is related to order statistics, e.g., [24,25]; however, such an approach leads to overly complicated analysis. We use the following approximation. The variables *x_i_* are assumed to be i.i.d. Gaussian but limited to the value of the maxima *z*. This is equivalent to conditioning the Gaussian distribution on |*x*| < *z*, omitting the index *i* to simplify presentation in the two following equations.

$$p\left(x\right)=\frac{\frac{1}{\sqrt{2\pi }}e^{-\frac{x^{2}}{2}}}{\int_{-z}^{z}\frac{1}{\sqrt{2\pi }}e^{-\frac{x^{2}}{2}}dx};\;\left|x\right|<z,\;p\left(x\right)=0\;\mathrm{otherwise}$$

So, the variance of *x_i_* is the following function of *z*:

(10) $$Var\left(z\right)=\frac{\int_{0}^{z}x^{2}e^{-\frac{x^{2}}{2}}dx}{\int_{0}^{z}e^{-\frac{x^{2}}{2}}dx}$$

**Figure 4:**
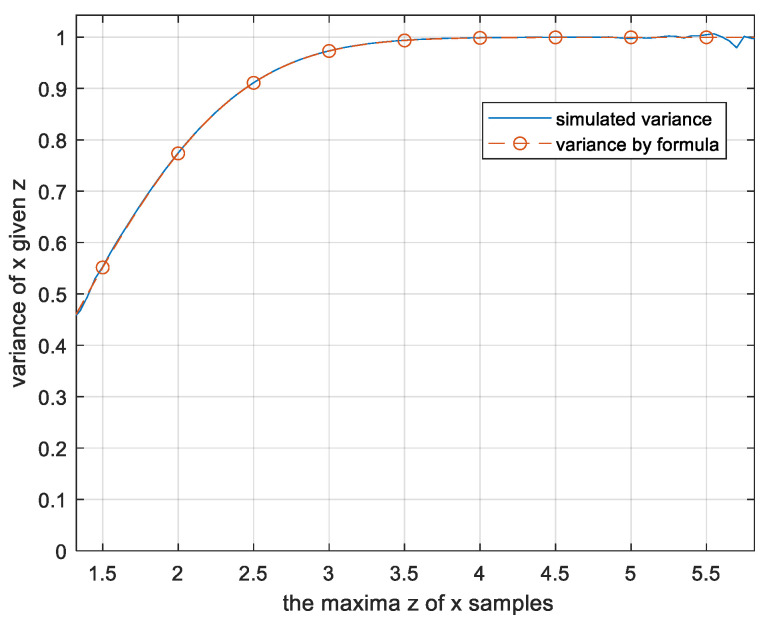
Variance of unordered *N* = 100 Gaussian samples *x_i_*, given their maxima *z* = max(|*x*_i_|) with the maximal sample excluded.

We verified (10) by simulation of *N*-tuples of Gaussian variables for *N* = 100 and found it accurate (see Figure 4).

The length *L* of the vector in the analysis below is not a random variable but, rather, the square root of the sum of the variances determined by *z*. For large *N* values, this is a good approximation and dropping the randomness forms a lower bound on the entropy, as explained below.

The length *L* of the normalized vector is then calculated, also accounting for the one sample for which |*x_i_*| = z:

$$L\left(z\right)=\sqrt{\frac{P\cdot Var\left(z\right)\cdot \left(N-1\right)}{z^{2}}+1}$$

We compute the volume *V_x_* of the peak-limited convex body with a unit power *P* = 1 using (8) and the PDF in (9).

(11) $$\;V_{x}=V_{N}^{u}\cdot E\left(L^{N}\right)=V_{N}^{u}\cdot \int_{0}^{\infty }p_{z}\left(z\right)L{\left(z\right)}^{N}dz$$

This is a lower bound since *L*(*z*) is assumed constant when *z* is given and only its root mean square is used. The accurate expression would replace in (11) the term *L*(*z*)*^N^* with *E*[*L*(*z*)*^N^*]. This would increase *V_x_* by Jensen’s inequality since *L*(*z*) is positive and raised to a high power in the last equation. Thus, the lower bound evaluated at *P* = 1 is

(12) $$V_{x}=V_{N}^{u}\cdot \int_{0}^{\infty }p_{z}\left(z\right){\left(\frac{Var\left(z\right)\cdot \left(N-1\right)}{z^{2}}+1\right)}^{\frac{N}{2}}dz$$

**Figure 5:**
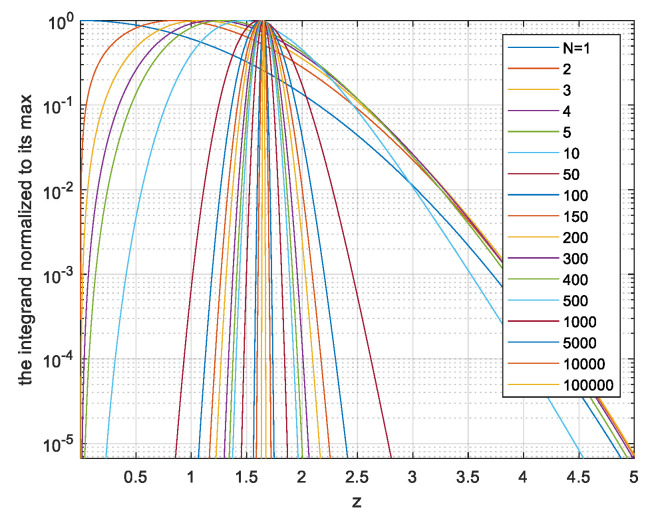
The integrand of Equation (12) for different values of *N*.

This is evaluated by numerical integration. The integrand of (12) indicates the range of the maxima z most contributing to the capacity. In Figure 5, we plot the integrand, normalized by its maxima, for each *N*.

**Figure 6:**
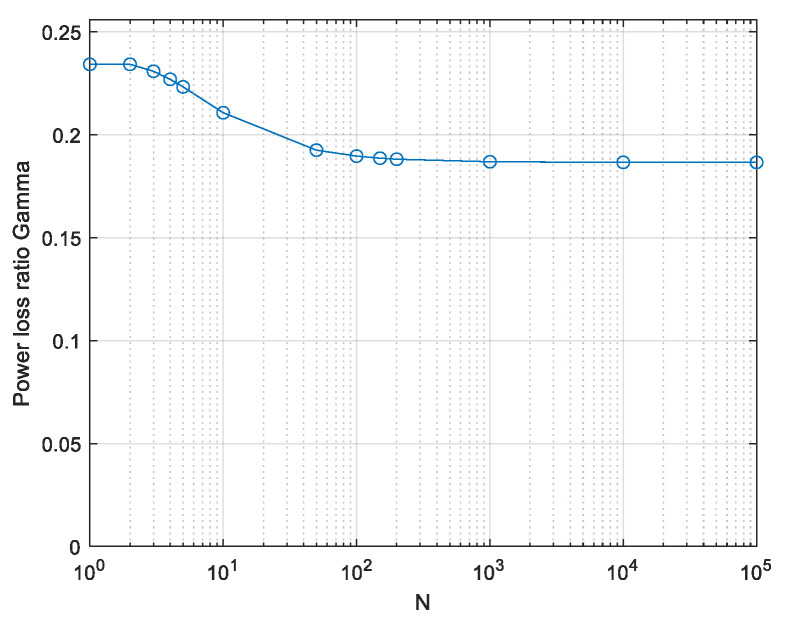
Computed lower bound on the power loss ratio γ; initial analysis limiting the power of Nyquist-rate samples only.

So, for *N* > 50, the system selects signals with relatively low peak values *z* of about 1.7 and not increasing much with the length *N* of the signal. This peak occurs, for *N* > 100, with extremely low probability, as seen in Figure 3. This implies that the signals contributing significantly to the integral (12) are rare among signals generated from independent Gaussian-distributed Nyquist-rate samples. The power loss ratio relative to the APL system is obtained by inserting the result of (12) into (7). The power loss is shown in Figure 6.

The initial analysis here, which peak-limits only the Nyquist-rate samples, addresses the same discrete symbols problem as [1,5,6], which present a power loss of γ = 0.2342 = 2/πe (see Appendix A). As explained above, the result in Figure 6 is a lower bound, which explains the gap from our result to the correct 2/πe. Only the values for very low *N* values approach the exact value. Still, the result is near enough to the exact one to allow qualitative understanding of the numerical evaluation presented further below.

The volume-based evaluation of capacity developed here is tight at asymptotically high SNRs as it is in the one-dimensional case [16] using the following. The capacity is

(13) $$C=\underset{p_{x}\left(x\right)}{\max}I\left(x;y\right)=h\left(y\right)-h\left(n\right)$$

Our lower bound in (3) and (4) has a higher value than $h\left(x\right)-h\left(n\right)$ (see the derivation in Appendix A); so, to show that it is tight it suffices to show that at high SNRs we have $h\left(x\right)≅h\left(y\right)$. The volume occupied by all vectors ***x*** is (11), which integrates the DFO of ***x***, denoted *L*, raised to the power of *N*, over all ***x***. For any ***x***, denoted ***x***^i^**,** the DFO is $L\geq \sqrt{P}$. Now, ***y*** cannot occupy a volume significantly larger than *V_x_* because the added noise increases the DFO in the direction of ***x***^i^ only by a small multiple of σ_n_, which is infinitely small relative to $\sqrt{P}$ at asymptotically large SNRs. The volume *V_y_* occupied by all vectors **y** enforces $h\left(y\right)\leq \log\left(V_{y}\right)$. Thus, $h\left(x\right)≅h\left(y\right)$ holds and the lower bound is tight at high SNRs.

### 3.3. Refinement to Continuous Signals

In this subsection, we replace the peak power limit on the Nyquist-rate samples **x** with the peak power limit on the whole continuous signal *x*(*t*). Prasad [26], in his equation (6.4), presents an authoritative and very convenient approximation to the PDF of the PAPR of a complex continuous signal with a duration of *N* Nyquist rate intervals *T*. It is derived from (6.3), which is the rigorous PDF of the PAPR of *N* Nyquist-rate samples, similar to our (9). Prasad carries this out by increasing *N* by a factor of α = 2.8, which is equivalent to replacing the infinity of correlated values in each Nyquist interval by α uncorrelated samples. This yields, in our case (9) of a real-valued signal, the continuous version of CDF and PDF of z_c_ = max_t_(|*x*(*t*)|)

(14) $$F_{Z_{c}}\left(z_{c}\right)={\left[F_{\left|X\right|}\left(z_{c}\right)\right]}^{\alpha N}$$

And

(15) $$p_{z_{c}}\left(z_{c}\right)=\alpha N{\left[1-2Q\left(z_{c}\right)\right]}^{\alpha N-1}\frac{2}{\sqrt{2\pi }}e^{-\frac{{z_{c}}^{2}}{2}}$$

**Figure 7:**
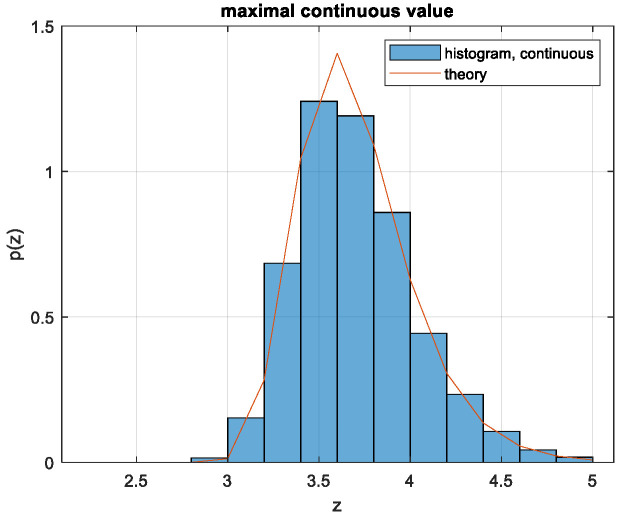
PDF of a maxima of a band-limited Gaussian unit power random process over N = 1001 Nyquist intervals. Histogram by simulation versus (15).

There are more advanced attempts to approximate $F_{Z_{c}}\left(z_{c}\right),$ such as [23]; however, those yielded still approximations and very involved expressions. The approximation (15) we use needs verification by simulation. We found the following α to be a good approximation: α = 2.3 for N = 101, α = 2.8 for N = 1001, α = 2.9 for N = 10,001 and N = 100,001. With N = 1001, α = 2.8 we obtain Figure 7.

**Figure 8:**
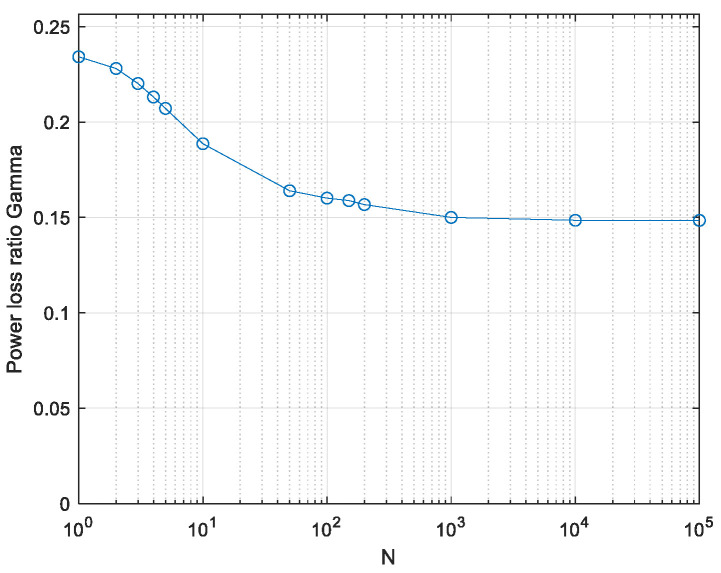
Computed lower bound on the power loss ratio γ, with continuous real-valued PPL signalling.

Plugging (15) into (12) and (7) yields the power loss due to the peak limit in Figure 8.

**Conjecture 1.**  *The power efficiency of 0.15 presented in Figure 8 is a lower bound on γ for continuous low-pass signals.*

**Explanation:**  *The only approximations used were (15), which is similar to [26] and verified numerically, and the approximation (10), verified numerically in Figure 4. Both the approximations seem sound. The result of 0.15 is a lower bound on γ as explained below (11).*

**Figure 9:**
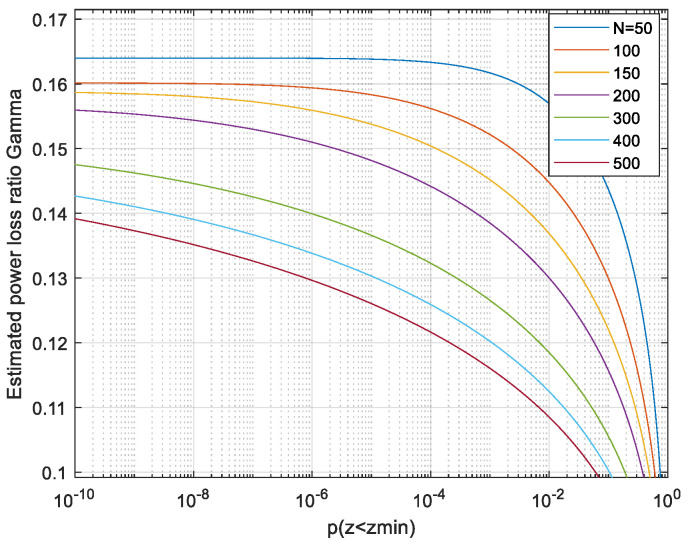
The ratio γ when vectors with a peak smaller then z_min_ are discarded.

In the next section, to avoid all the approximations used for the analysis above, we shall evaluate the volume *V*_x_, instead of the statistical expectation (11), by generating the vectors ***x*** at random and estimating *V*_x_ by averaging via (8). We learn from the analysis the following lessons on the number of vectors required in the Monte Carlo evaluation. For N > 100, the main contribution to *V*_x_ and to capacity is from not overly high values of *z* which have very low probabilities, as seen by combining Figure 5 with Figure 7. That is, the rare vectors the peak power of which is not too high contribute most to *V*_x_. Generating at random enough vectors, the probability of which is very low, requires a sufficient number of random vectors. As seen in Figure 3 and Figure 7, for *N* > 100, the PDF *p*(*z*) of a maxima of Gaussian process decreases sharply with decreasing *z*. So, it is of interest to evaluate the power ratio γ with the volume in (12) integrated only over *z* > *z_min_* and plotting it as a function of *p* (*z* < *z_min_*). Such a plot will enable us to assess the number of simulated sequences required for convergence as follows: select a value of *z_min_* that is small enough to enable evaluating γ correctly using Figure 9, in which *p* (*z* < *z_min_*) is presented rather than *z_min_*, and use number of vectors larger than 1/*p* (*z* < *z_min_*).

Thus, for *N* = 50 about 10^4^ simulated sequences are required; for *N* = 200, more than 10^8^ should be used. With 10^7^ simulated vectors, we can expect reliable results up to an *N* of about 100. To reach a reliable result, we need to simulate enough vectors to be on the horizontal section of the curve. A useful criterion can be stable results under ten-fold change in p (*z* < *z_min_*). A practical method to examine this is discarding the 10 most contributing vectors and permitting only a small change in γ. When using the Monte Carlo evaluation with a too-large *N*, the estimates will be lower than the true values because the low- probability vectors which contribute most to the capacity will be missed.

## 4. Monte Carlo Evaluation

**Figure 10:**
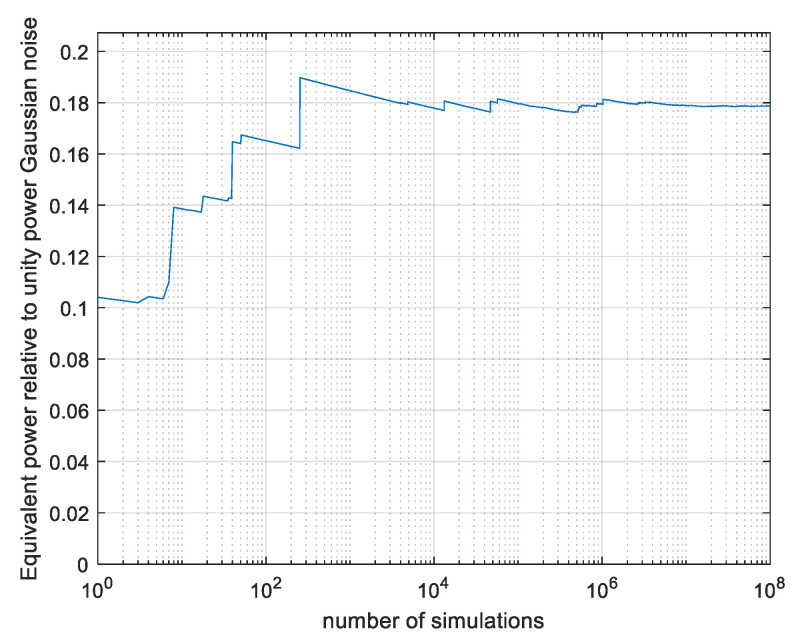
Monte Carlo-evaluated power loss ratio γ; CP-FDE signalling, N = 101, as a function of number of simulated vectors *N_sim_*.

The Monte Carlo estimation avoids all the approximations used in the analysis section by generating the vectors ***x*** at random and estimating the expectation by averaging as explained above. The evaluation uses importance sampling as presented in Appendix B to accelerate convergence. The signals are evaluated as to be compatible with CP-FDE signalling, that is, generating *N* Nyquist-rate samples at random, oversampling while keeping the sequence duration at *NT*, performing Fast Fourier Transform (FFT), brick-wall filtering in the frequency domain, and IFFT. This is the classical transmit side processing of CP-FDE prior to adding the cyclic prefix. Each sequence is scaled as to have max(|*x*(*t*)|) = 1; the length *r* of the scaled and sampled ***x*** is calculated and processed by the Monte Carlo equivalent of (8). As a verification, the discrete symbols case, that is, power limiting only the Nyquist-rate samples and not the signal in between, as in [16], evaluates immediately to the correct γ = 2/πe. To evaluate our continuous system, an oversampling ratio of 30 samples per Nyquist interval is used. We begin with a sequence length of N = 101, which is predicted to converge well with the analysis. The results are plotted in Figure 10 as a function of the number *N_sim_* of simulated vectors.

**Figure 11:**
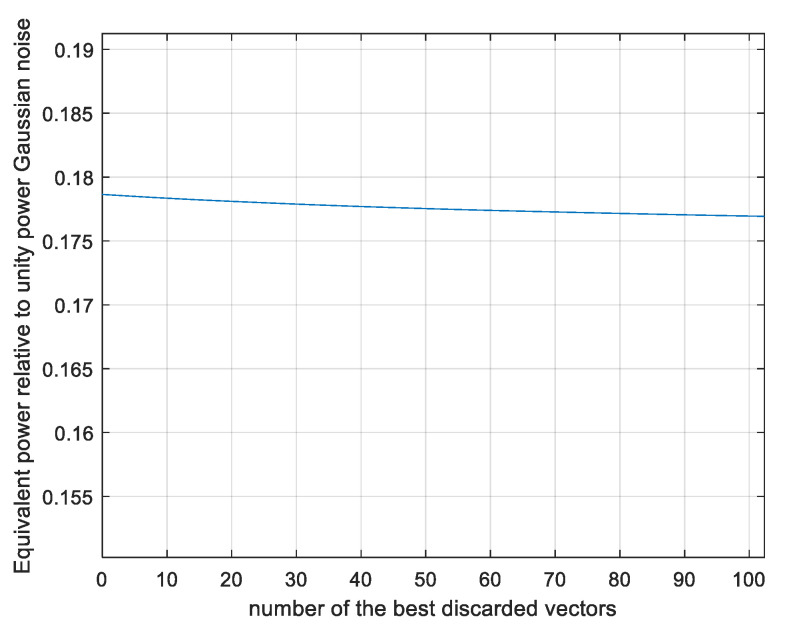
Monte Carlo-evaluated power loss ratio γ; N = 101, as a function of number of the most contributing vectors discarded.

The result of γ = 0.18 is somewhat larger than the lower bound of 0.15 computed by analysis in Figure 8, and there is a convergence after 10^6^ simulations. The convergence roughly follows the expectations based on Figure 9. For example, at 10^2^ simulations, γ is about 0.02 below its final value as it is approximately for *p* (*z < zmin*) = 10^−2^ in Figure 9. In Figure 11, we present the results with the same simulation runs if the most contributing runs out of the total 10^8^ are discarded.

**Figure 12:**
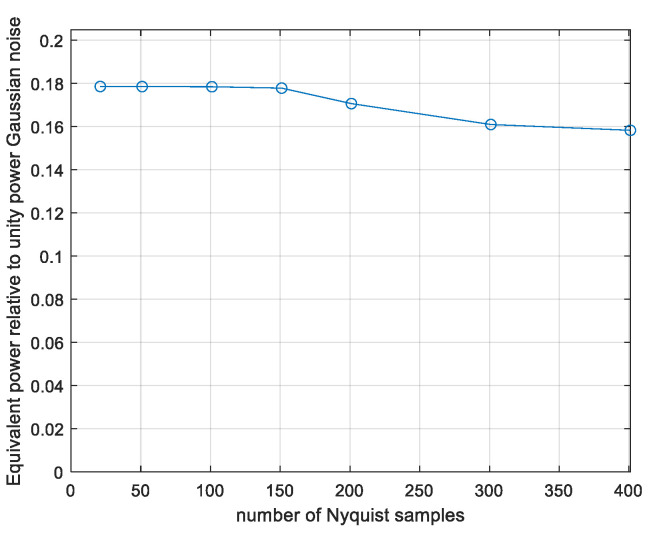
Monte Carlo evaluated power loss ratio γ, CP-FDE signalling, as a function of number of signal duration in Nyquist intervals *N*.

As seen, discarding the ten most contributing runs degrades the performance less than 0.2% and discarding 100 vectors degrades it by 1%, indicating a reliable convergence. This is comparable to the rough expectation based on Figure 9. Next, we estimate *γ* as a function of the sequence length *N* using *N_sim_* = 10^8^ simulated vectors per point. The results are plotted in Figure 12.

**Figure 13:**
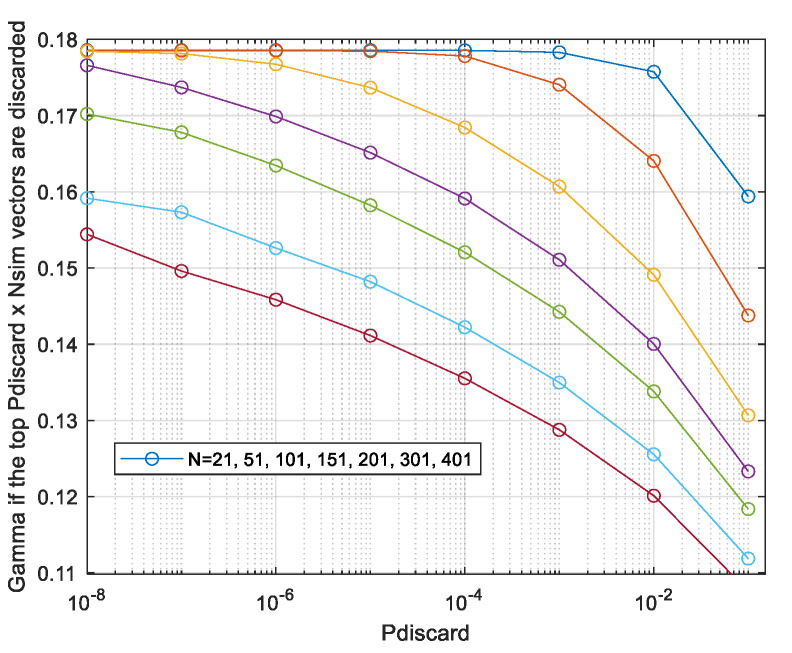
Monte Carlo-evaluated power loss ratio γ; CP-FDE signalling, if the most contributing vectors the probability of which is *Pdiscard* are discarded. The lines correspond to different values of *N*, increasing from top to bottom of the figure.

The γ is above 0.177 for *N* up to 101 and then starts to decrease. In Figure 13, we show the performance when discarding the *p* (discard) × *N_sim_* most contributing runs.

**Figure 14:**
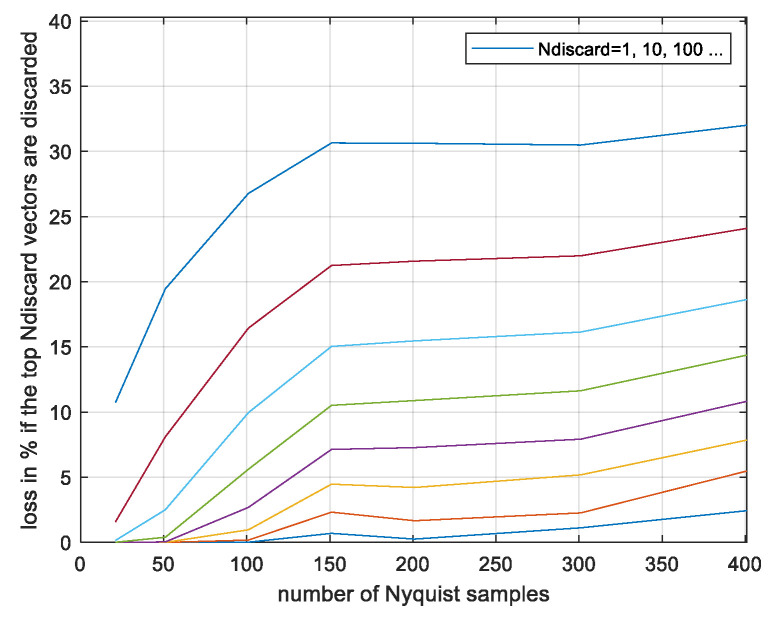
Monte Carlo-evaluated power loss ratio γ; CP-FDE signalling, if the most contributing *Ndiscard* vectors are discarded. The lines correspond to different values of *Ndiscard* increasing fom bottom to top of the figure.

The behaviour is qualitatively as predicted in Figure 9, that is, the decrease in the estimated results with rising *N* is partly due to an insufficient number of simulation runs, with the most contributing vectors becoming rarer as *N* rises. And, for lengths up to 101 Nyquist intervals, the evaluation is well converged, that is, the results remain the same even if the 10 most contributing runs are discarded as seen in Figure 14.

## 5. Bandpass Signals

We extend the analysis to bandpass signals represented in the complex-valued baseband. We reuse the previous notation with modifications as follows. The encoder produces a complex-valued low-pass signal *x*(*t*) in the frequencies |*f*| < 0.5*B*. The noise is complex-valued with power spectral density *N*_0_ (two-sided). The signal is peak-limited, that is, $\left|x\left(t\right)\right|\leq \sqrt{P}\;\mathrm{for}\;\mathrm{all}\;t$. The Signal to Noise ratio is defined as $\rho =\frac{P}{BN_{0}}\;.$ The equations are updated as follows (see Appendix A). The classical capacity per Nyquist-rate sample of the APL channel is

(16) $$C_{a}=\log_{2}\left(\frac{P}{N_{0}B}+1\right)$$

The lower bound γ on the power loss ratio of the PPL channel relative to the APL channel remains

(17) $$\gamma =\frac{P^{e}}{P}\leq 1$$

where *P*^e^ denotes the entropy power of ***x*** defined now as

(18) $$\;P^{e}=\frac{1}{\pi e\;}\;\cdot \;e^{\frac{1}{N}h\left(x\right)}$$

It is shown in Appendix A that the following holds:

(19) $$C\geq \log_{2}\left(\frac{\gamma \cdot P}{N_{0}B}+1\right)$$

In the PPL complex-valued channel, the power ratio γ is shown in Appendix A to be

(20) $$\;\gamma =\frac{V_{x}^{\frac{1}{N}}}{\pi e}$$

evaluated with *P* = 1.

To compute the upper bound on capacity by power-limiting only the Nyquist-rate samples and not the signal in between, each complex sample of the entropy-maximizing distribution is uniformly distributed over a disk with a radius of $\sqrt{P}$ yielding $V_{x}={\left(\pi {\sqrt{P}}^{2}\right)}^{N}={\left(\pi P\right)}^{N}$, and the ratio γ which is both an upper bound for our continuous signals case and an accurate value for the discrete power-limited problem in [27] is

(21) $$\gamma =\frac{1}{e}\;,$$

See Appendix A. This value falls correctly between the Smith-based lower and upper bounds in Figure 2 of [27], thus tightening the lower bound of [27] at high SNRs.

Denote the maxima of |*x*(*t*)| over *N* Nyquist intervals as z and *w* = z^2^. The work in [26] reminds us that *w*_s_=|*x*(*t*)|^2^ is central chi-square distributed with two degrees of freedom (scaled to a unity mean) with $p_{w_{s}}\left(w_{s}\right)=e^{-w_{s}}$ and then provides a good approximation verified by simulations on the CDF of w:

(22) $$\;F_{w}\left(w\right)={\left(1-e^{-w}\right)}^{\alpha N}$$

with α = 2.8. By differentiation,

(23) $$p_{w}\left(w\right)=\alpha N{\left(1-e^{-w}\right)}^{\alpha N-1}e^{-w}$$

Equation (10) is replaced by

(24) $$E\left(w\right)=\frac{\int_{0}^{w}w_{s}e^{-w_{s}}dw_{s}}{\int_{0}^{w}e^{-w_{s}}dw_{s}}$$

where *E* denotes expectation. The last equation was verified numerically by simulation of *N*-tuples of *w_s_* variables with *N* = 100; see [19] for a plot of the results. The typical length *L* of the vector normalized to unity max(|*x*(*t*)|) will be, then,

(25) $$L\left(w\right)=\sqrt{\frac{E\left(w\right)\cdot \left(N-1\right)}{w}+1}$$

The volume *V_x_* is now $V_{2N}^{u}\cdot E\left(L^{2N}\right)$ because the signals are complex, which doubles the dimension of the convex body. The volume *V_x_* of the peak-limited convex body with a unit power *P* = 1 using the PDF *p_w_* in (23) is

(26) $$V_{x}=V_{2N}^{u}\cdot \int_{0}^{\infty }p_{w}\left(w\right)L{\left(w\right)}^{2N}dw$$

**Figure 15:**
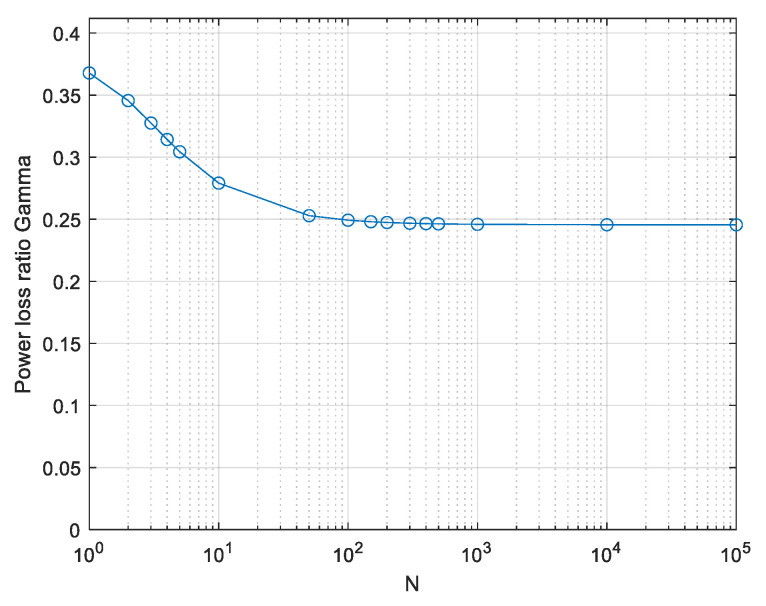
Computed lower bound on power loss ratio γ; continuous PPL signalling. Complex-valued signals.

This is a lower bound as in the real-valued case. It is evaluated by numerical integration and the power loss ratio relative to the APL system is evaluated by (20). In the discrete symbols scenario, that is, power-limiting the Nyquist-rate samples only, evaluated by using α = 1 in (23), the exact value is $\frac{1}{e}≅0.368$. It is reached only at very low values of *N* and decreases at larger *N* values similarly to the real signals case (see the plot in [19]). The lower bound on power efficiency γ with continuous peak power limiting, using α = 2.8, is plotted in Figure 15.

**Conjecture 2.**  *The power efficiency of 0.245 presented in Figure 15 is a lower bound on γ for continuous bandpass signals.*

**Explanation:**  *The only approximations used were (22), adopted from [26], and the truncation in (24), which was verified numerically. Both the approximations seem sound. The result of 0.245 is a lower bound on γ as explained below (11).*

**Figure 16:**
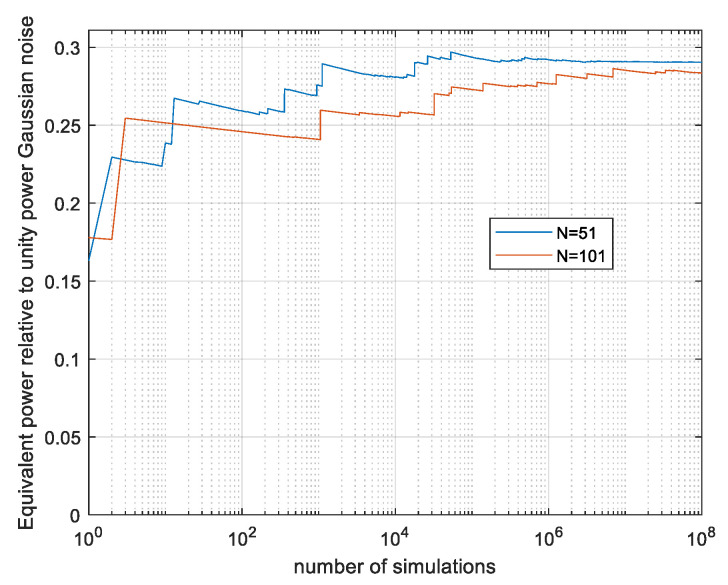
Monte Carlo-evaluated power loss ratio γ; CP-FDE signalling, with *N* = 51 and 101 as a function of number of simulated vectors. Complex-valued signals.

The Monte Carlo estimation, as in the real-valued case, avoids all the approximations used in the analysis. The signals are evaluated so as to be compatible with CP-FDE signalling. As a verification, the discrete symbols case evaluates immediately to the correct γ = 1/e. To evaluate our continuous system, an oversampling ratio of 30 samples per Nyquist interval is used. We begin with sequence lengths of 51*T* and 101*T* predicted to converge by the analysis. The results are shown in Figure 16 as a function of the number of simulated vectors *N_sim_*.

**Figure 17:**
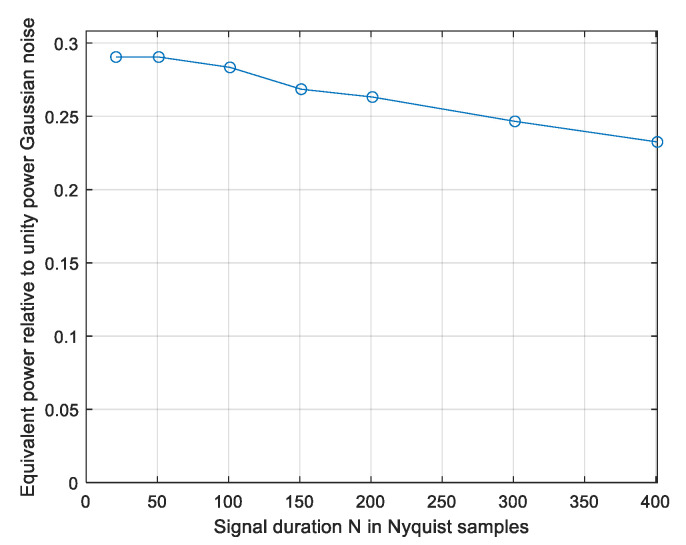
Monte Carlo-evaluated power loss ratio γ; CP-FDE signalling as a function of number of signal durations in Nyquist intervals *N*. Complex-valued signals.

The results N = 51, γ = 0.29 and N = 101, γ = 0.284 are slightly larger than the computed lower bound of 0.245 in Figure 15, and there is a convergence after 10^6^ and 10^7^ simulations for N = 51 and N = 101, respectively. Next, we estimate γ as a function of the sequence length *N* using *N_sim_* = 10^8^ vectors per point. The results are presented in Figure 17.

To assess convergence, figures similar to Figure 9 and Figure 11 and Figure 13 and Figure 14 were constructed for the complex-valued signals; those are available in [19]. The behaviour is similar to the real-valued case. It is evident that more vectors are required in the complex case, e.g., *N* = 50 requires about 10^5^ vectors while N = 101 requires about 10^8^. The decrease in the estimated results with growing *N* values is in part due to not enough simulation runs, with the most contributing vectors becoming rarer as *N* rises. The *N* = 51 result γ = 0.29 is reliable and is stable even if the 100 most contributing vectors are discarded. The result at N = 101 is probably slightly lower than the true γ; it loses 1.6% if the 10 most contributing vectors are discarded. The result γ = 0.29 is the γ of the CP-FDE signalling at a signal duration of 51*T* and the values in Figure 17 at *N* > 100 are lower bounds applicable to the CP-FDE signalling.

## 6. Conclusions

The important problem of the capacity of the PPL channel was investigated. We focussed on the power efficiency γ, which provides a lower bound on capacity, tight at asymptotically high SNRs. The results are summarized in Table 1. We showed that the new lower bounds on γ are about 3.3 and 8.6 times higher than previously known in the low-pass and in the bandpass cases, respectively. The gap to the upper bounds is narrowed to less than 2 dB. The numerical results for γ are valid for practical CP-FDE signalling with limited transmission duration, while the general analytical results are lower bounds on γ as explained below (11) and rely on two approximations, rendering them conjecture. The lower bounds based on γ via (3) are tight at high SNRs and show that the peak power constraint causes, at high SNRs, power loss of about 6 dB in the bandpass case and a little more in the low-pass case.

**Table 1:** Bounds on power efficiency γ of peak-limited signals.

The Bound	Results of [5]	Results of [6]	This Work, General Result, But Remains Conjecture	This Work	This Work, CP-FDE Signalling, Duration of 101 Nyquist Intervals
**Low-pass lower bound**	0.0361 = π/32e	0.04470	γ > 0.15		γ = 0.18
**Low-pass upper bound**	0.2342 = 2/πe Also presented in [1].	0.2342 = 2/πe			
**Bandpass lower bound**	$\frac{\pi^{2}}{128e}\;$= 0.0284		γ > 0.245		γ = 0.284
**Bandpass upper bound**				$\frac{1}{e}≅0.368$	

Future work: The above-stated upper bounds are based on peak-power limiting of only the Nyquist-rate samples which are assumed to be independent. It would be interesting to also account for the correlation which is implied on these samples, as is reflected in [15], to sharpen the upper bounds. This might be addressed via a Multiple Input Multiple Output (MIMO) structure, where both constraints should be addressed. The MIMO setting might also be relevant for a super Nyquist sampling, again accounting for the fact that all samples are peak-power limited; see [15,28,29] for relevant results that might be used.

Also, our analysis showed that the capacity achieving signals are a small proportion of Gaussian-bandlimited signals and this proportion vanishes with growing signal duration. The same holds for codewords of error correcting codes; the codewords are a vanishing proportion of all random binary sequences. The peak limit selects the appropriate signals resembling somewhat the effect of the parity check matrix of a binary error correcting code. Thus, further work should seek structures of PPL signals approaching the channel capacity similar to the vast work performed in recent decades on error correcting codes.

## Data Availability

Additional figures are available in [19].

## Appendix A. Bounds

We summarize the bounding technique used, e.g., in [10] for completeness. We shall start with real-valued signals. We denote differential entropy as *h*. The channel is an AWGN channel with scalar-valued real symbols (1). The noise has a power of $\sigma_{n}^{2}=N_{0}B$ and a per-sample entropy of $h\left(n\right)=0.5\log\left(\sigma_{n}^{2}\cdot 2\pi e\right)$. The capacity per sample is

$$C=\frac{1}{N}\left[h\left(y\right)-h\left(y|x\right)\right]=\frac{1}{N}\left[h\left(y\right)-h\left(n\right)\right]$$

where the bold symbols denote vectors and *N* is the number of Nyquist-rate samples.

Using the EPI,

(A1) $$\;e^{\frac{2}{N}h\left(y\right)}\geq e^{\frac{2}{N}h\left(x\right)}+e^{\frac{2}{N}h\left(n\right)}$$

$$h\left(y\right)\geq \frac{N}{2}\log\left(e^{\frac{2}{N}h\left(x\right)}+e^{\frac{2}{N}h\left(n\right)}\right)$$

and

$$e^{\frac{2}{N}h\left(n\right)}=\sigma_{n}^{2}\cdot 2\pi e$$

So, the per-sample capacity *C* is

$$C\geq \frac{1}{2}\log\left(e^{\frac{2}{N}h\left(x\right)}+\sigma_{n}^{2}\cdot 2\pi e\right)-\frac{1}{2}\log\left(\sigma_{n}^{2}\cdot 2\pi e\right)$$

(A2) $$C\geq \frac{1}{2}\log\left(\frac{\frac{1}{2\pi e\;}\;\cdot \;e^{\frac{2}{N}h\left(x\right)}}{\sigma_{n}^{2}\cdot }+1\right)$$

We use the entropy power of **x** definition $P^{e}=\frac{1}{2\pi e\;}\;\cdot \;e^{\frac{2}{N}h\left(x\right)},\;$yielding, in bits per sample,

(A3) $$C\geq \frac{1}{2}\log_{2}\left(\frac{P^{e}}{\sigma_{n}^{2}}+1\right)$$

followed by the pre-SNR factor (3), (4). The ratio γ in (4) is the lower bound on the power loss ratio of the PPL channel relative to the APL channel and is valid at all SNRs. It is used in [6] as the pre-SNR factor. Now, *h*(*x*) = log(*V_x_*), where *V_x_* is the volume of the convex body if the distribution of ***x*** is the entropy-maximizing uniform one. This yields (6). It follows from the peak power limit *P* that *V_x_* is proportional to $P^{\frac{N}{2}}$. Then, by (6)*, P^e^* is linearly proportional to *P* and volume-derived γ is invariant with respect to *P*. Therefore, γ (4) can be evaluated with *P* = 1 with no loss of generality.

To provide an upper bound on γ, the peak power limit can be applied to the Nyquist-rate samples only and not to the signal in between as conducted in [5]. In this case, the signal samples are limited to $-\sqrt{P}<x<$ $\sqrt{P}$, the convex body is an *N*-cube with volume (2$\sqrt{P}$)*^N^* and (7) yields the power loss γ of $\frac{2}{\pi e}$ presented in [5].

**Extension to complex-valued signals:**

The symbols are complex. The real and imaginary components of the noise have a power of $\sigma_{n}^{2}$/2 = *N_0_B*/2 each and the per-symbol noise entropy is $h\left(n\right)=\log\left(\sigma_{n}^{2}\cdot \pi e\right)$. The per-symbol capacity is

$$C=\frac{1}{N}\left[h\left(y\right)-h\left(n\right)\right]$$

The EPI formula as in (A1) holds for *N*-dimensional real vectors; for *N*-dimensional complex vectors, it is

$$e^{\frac{1}{N}h\left(y\right)}\geq e^{\frac{1}{N}h\left(x\right)}+e^{\frac{1}{N}h\left(n\right)}$$

yielding

(A4) $$C\geq \log\left(\frac{\frac{1}{\pi e\;}\;\cdot \;e^{\frac{1}{N}h\left(x\right)}}{\sigma_{n}^{2}}+1\right)$$

For a Gaussian signal with power *P*, this turns into the familiar (16). We can denote the entropy power *P^e^* of the complex-valued x as in (18) and define γ by (4) yielding (19). As in the real-valued case, the maximal entropy is $h\left(x\right)=\log\left(V_{x}\right)$, where *V_x_* is the volume of the convex body. This yields, for the uniform entropy-maximizing distribution of ***x***,

(A5) $$P^{e}=\frac{1}{\pi e}V_{x}^{\frac{1}{N}}$$

and

(A6) $$C\geq \log\left(\frac{\frac{1}{\pi e}V_{x}^{\frac{1}{N}}}{\sigma_{n}^{2}\cdot }+1\right)$$

and (20) follows.

## Appendix B. Importance Sampling in the N-Cube

As explained, generating signals at random on the *N*-sphere yields Gaussian-distributed samples. This enables analytical results and straightforward simulation; however, there is a problem. As explained above, with no oversampling, the optimal signal is distributed uniformly in the hypercube, the signal volume is concentrated near the *N*-cube corners and the corner regions are visited by the random signals, which are uniform on the *N*-sphere, very rarely. Thus, the evaluation by simulation converges very slowly.

We shall accelerate the estimation by importance sampling, generating the random signals uniformly in the hypercube instead on the unit-radius hypersphere. This accelerates the evaluation to some degree even if the signal region in the continuous case is definitely not a hypercube. The principles are as follows:

The signals are generated uniformly distributed in the volume of an *N*-cube by randomly generating each sample *x_i_* uniformly in the [5] interval. For each signal instance, a probability factor *P_c_* is computed, equal to probability of this particular angle if the signals were generated uniformly over angles, that is, uniformly over the unity radius *N*-sphere, divided by the probability if the signals were generated uniformly in the *N*-cube. *P_c_* is the ratio of the volume of the hyperball enclosed in an infinitesimal angle sector around the selected signal to the volume of the hypercube enclosed in the same-width angle sector corrected by the volume ratio of the hypercube 2*^N^* and the hypersphere $V_{N}^{u}$. Thus, denoting by *L_c_* the DFO of the hypercube surface in the chosen direction,

$$P_{c}=\frac{1}{L_{c}^{N}}\frac{2^{N}}{V_{N}^{u}}$$

The volume evaluation (8) is modified according to the importance sampling method to

(A7) $$V_{Z}=E_{\theta }\left(V_{N}^{u}\cdot r^{N}\cdot P_{c}\right)=E_{\theta }\left(V_{N}^{u}\cdot r^{N}\cdot \frac{1}{L_{c}^{N}}\frac{2^{N}}{V_{N}^{u}}\right)=E_{\theta }\left\{{\left(\frac{2\cdot r}{L_{c}}\right)}^{N}\right\}$$

where the expectation is carried with signals distributed uniformly over the N-cube.

Intuitive check: suppose the signal body is an *N*-cube with samples −1 < x_i_ < 1. Then, *r = L_c_* is always the case since *r* is scaled by the peak limiting to touch the hypercube surface and the volume will be 2*^N^* as it should be.

**Extension to the complex-valued case:**

The signals are now generated uniformly distributed in the volume of a 2*N*-dimensional body by randomly generating each of the *N* complex samples *x_i_* independently and uniformly over an area in the complex plane enclosed by a circle with a unity radius reflecting |*x_i_*| < 1 and an area of π.

The last two equations are updated accordingly accounting for the volume of the 2*N*-dimensional body of $\pi^{N}$ and for the number of dimensions being 2*N* since each *x_i_* is complex. *L_c_* is the DFO of the surface of the 2*N*-dimensional body in the chosen direction.

$$P_{c}=\frac{1}{L_{c}^{2N}}\frac{\pi^{N}}{V_{2N}^{u}}$$

The volume evaluation (8) is modified according to the importance sampling method to

(A8) $$V_{Z}=E_{\theta }\left(V_{2N}^{u}\cdot r^{2N}\cdot P_{c}\right)=E_{\theta }\left\{\pi^{N}{\left(\frac{r}{L_{c}}\right)}^{2N}\right\}$$

## Appendix C. Symbols, Variables and Abbreviations

**Symbol or Abbreviation**	**Meaning**
APL	Average Power Limited
AWGN	Additive White Gaussian Noise
*B*	Bandwidth
***b***, bold italic letters	Vector of Samples of *b*(*t*)
*C*	Capacity
*C_a_*	Capacity Over the APL Channel
CDF	Cumulative Distribution Function
CGC	Constrained Gaussian Channel
CP-FDE	Cyclic Prefix-Assisted Frequency Domain Equalization
DFO	Distance From the Origin
DPD	Digital Pre-Distortion
EPI	Entropy Power Inequality
*E* *_θ_*	The Statistical Expectation with Respect to *θ*
*f*	Frequency
*F*	Cumulative Distribution Function
FDMA	Frequency Domain Multiple Access
FFT	Fast Fourier Transform
*h*	Differential Entropy
*I*(*x;y*)	Mutual Information
i.i.d.	Independently and Identically Distributed
IFFT	Inverse Fast Fourier Transform
*L*	Length of the Signal Vector *x*
Log	The Natural Logarithm
LTE	Long-Term Evolution
MIMO	Multiple Input Multiple Output
MUI	Mutual Information
*N*	Duration of Message in Nyquist Intervals
*n*(*t*)	White Gaussian Noise
*N*_0_	Noise Power Spectral Density
*N_sim_*	Number of Simulated Vectors
*P*	Power and Peak Power
PAPR	Peak to Average Power Ratio
*P_c_*	Probability factor in Importance Sampling
PDF	Probability Density Function
*P^e^*	The Entropy Power
PPL	Peak-Power Limited
PSD	Power Spectral Density
*p_x_*(*x*) or *p*(*x*).	Probability Density Function of *x*
*Q(x)*	The Q-function
*r*	Distance From the Origin
*R^N^*	The *N*-Dimensional Vector Space Of Real Variables
SC-FDMA	Single-Carrier FDMA
SNR	Signal to Noise Ratio
$S_{N-1}^{u}\;$	The Area of the Unit *N* − 1 Sphere
*t*	Continuous Time
*T*	Nyquist Interval
*Var*	Variance
$V_{N}^{u}$	The Volume of the *N*-Dimensional Ball with Unit Radius
*V_x_*	Volume Occupied by all Permissible Vectors ***x***
*x* = (*x*_1_ *… x_n_ … x_N_*)	Vector of Nyquist-Rate Samples of *x(t)*
*x(t)*	Continuous Input Signal
*y(t)*	Continuous Output Signal
*z*	Maximal Abs. Value of the Nyquist-Rate Samples of *x*(t)
z_c_	Maximal Absolute Value of *x(t)*
*α*	Correction Factor by Prasad
*γ*	Lower Bound on the Power Loss Ratio
*θ*	Angles from the Origin to Points on the Surface of the Convex Body
*ρ*	Signal to Noise Ratio
*σ*_n_^2^	Noise power
